# Marine and Coastal Science: U.S. Ocean Policy Report Card

**DOI:** 10.1289/ehp.114-a216

**Published:** 2006-04

**Authors:** John Tibbetts

If the U.S. government were a student, it would be on the verge of flunking Ocean Policy 101, to judge by the *U.S. Ocean Policy Report Card* issued 3 February 2006 by the Joint Ocean Commission Initiative. The United States has accomplished far too little in response to a health crisis in the nation’s oceans, coasts, and Great Lakes, according to the report card. More than a dozen federal agencies have a say in setting and implementing ocean policy, and they often fail to work together. Policy makers need to repair this fragmented system of ocean governance, and they need to do it fast.

The 10-member initiative was created to track government response to two landmark ocean commissions. In September 2004, the U.S. Commission on Ocean Policy, a presidential panel chaired by retired admiral James Watkins, recommended that Congress boost ocean research funding, improve fisheries management, and consolidate federal oversight over ocean policy. A year earlier, the privately funded Pew Oceans Commission, chaired by Clinton White House chief of staff Leon Panetta, had reached many of the same conclusions.

Yet policy makers have done too little to fix the nation’s confusing system of authority over coastal and marine ecosystems, says initiative member Andrew Rosenberg, a fisheries scientist at the University of New Hampshire. “A lot of the issues that the commissions talked about came true with Hurricane Katrina: wetlands loss, lack of planning, inadequate infrastructure, risk from natural hazards, continued fragmentation of ocean policy, major damage to fisheries in the [Gulf of Mexico],” he says. “We need a lead ocean agency to take a new direction internally and to cooperate with other agencies to consolidate and focus programs.”

Initially, government leaders responded positively to the two commission reports, a response that earned an A– on the report card. In December 2004, Bush released an action plan that included the creation of a committee to oversee ocean policies (this committee will release a priorities plan and implementation strategy at the end of 2006). Congress and state governors also acknowledged the major recommendations of both commissions.

But momentum has stalled since then. Congress and the Bush administration have not done enough to create a national ocean policy and to strengthen NOAA to the point that it can serve as the lead ocean agency, according to the report card, which gives leaders a D+ in the category of national ocean governance reform.

Policy makers, moreover, receive an F in new funding for ocean policy and programs. The U.S. Commission on Ocean Policy called for a doubling over five years of ocean-related research funding. But federal ocean research received level funding in fiscal year 2006, and Bush’s 2007 budget proposal would reduce NOAA’s oceanic and atmospheric research support by 9%, to $338 million. Important ocean programs such as the six NOAA Undersea Research Centers were severely cut for 2006 and would not be fully restored under the 2007 budget.

“We are very disappointed in the president’s budget request for NOAA,” says Ted Morton, federal policy director of the international nonprofit Oceana. “We were expecting to see more increases for critical ocean and coastal programs. The commissions were clear that significant increases were necessary to restore ocean health.”

The report card gives government a C+ for fisheries management reform, noting the Senate’s establishment of bipartisan support in an effort to reauthorize the 1976 Magnuson-Stevens Fisheries Conservation and Management Act, the primary federal law governing fisheries management.

Another bright spot is the ongoing reform of ocean management occurring in some states and regions. Among the most notable are efforts in California, Puget Sound, and the Gulf of Maine. These management efforts, which earned a B–, are using a broad ecosystem approach and include government at all levels. The idea is to work toward regional cooperation and across jurisdictional lines in new and existing programs. In the Pacific Northwest, for example, the Northwest Straits Commission addresses marine conservation in the region, the Shared Strategy for Puget Sound addresses salmon recovery in that water body, and the Puget Sound/Georgia Basin International Task Force addresses environmental problems associated with population growth.

## Figures and Tables

**Figure:**
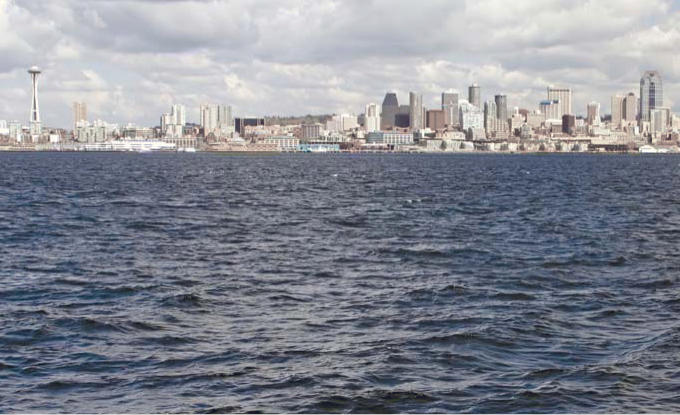
Bright spot in a dim picture. Although some regions such as Puget Sound (above) are earning good marks for ocean management, overall the United States is failing miserably when it comes to the nation’s ocean policies, according to a recent report by the Joint Ocean Commission Initiative

